# From Discovery to Diagnostic Reasoning: Research, Education, and Clinical Scholarship

**DOI:** 10.51894/001c.167936

**Published:** 2026-08-21

**Authors:** Francis Akenami, Rana Ismail, Andrea Amalfitano

**Affiliations:** 1 College of Osteopathic Medicine – Research, Innovation, and Scholarly Engagement (RISE) Michigan State University, East Lansing, MI, USA; 2 College of Osteopathic Medicine – Graduate Medical Education Alliance Michigan State University, East Lansing, MI, USA

**Keywords:** Clinical Scholarship, Diagnostic Reasoning, Translational Research, Medical Education, Quality Improvement, Global Health, Osteopathic Medicine, Case Reports

## Abstract

The Spartan Medical Research Journal (SMRJ) presents this special symposium issue highlighting scholarly work from the Fourth Annual Research Day of the Michigan State University College of Osteopathic Medicine (MSUCOM), held May 12, 2026, in Novi, Michigan. Supported by the MSUCOM Dean’s Discretionary Fund, the Graduate Medical Education Alliance, and Research, Innovation, and Scholarly Engagement (RISE), the annual event reflects an institutional commitment to fostering inquiry, mentorship, and the development of future physician-scientists. More than 200 submissions were received from MSUCOM medical students and GME Alliance-affiliated residents, with approximately 180 selected for oral or poster presentation following review by the MSUCOM Research Day Planning Committee. This special issue presents 131 published abstracts, encompassing basic and translational science, clinical research, case reports, quality improvement, medical education, global health, and diagnostic scholarship. Collectively, these contributions demonstrate the breadth of trainee-led investigation and highlight recurring themes of translational discovery, diagnostic reasoning, interdisciplinary collaboration, educational innovation, and improvement in patient care. Although conference abstracts represent concise and often preliminary accounts of scholarly work rather than definitive analyses, they provide an important forum for disseminating emerging observations, stimulating scientific discussion, and fostering collaboration. We commend the student and resident investigators and their mentors for their contributions and encourage continued development of these projects into rigorous, full-length scholarly publications.

## INTRODUCTION

The 2026 Spartan Medical Research Journal (SMRJ) Research Day special issue brings together 131 published abstracts spanning basic and translational science, clinical research, quality improvement, medical education, global health, and case-based learning. The collection reflects the breadth of scholarship generated across graduate medical education and affiliated academic settings, while illustrating how discovery, bedside observation, educational inquiry, and systems improvement can inform one another.

For a collection of this scale, the most useful editorial is thematic rather than sequential. The aim is not to catalogue every abstract sentence by sentence, but to identify the questions investigators are asking, the clinical and educational problems they are attempting to solve, and the values connecting work across disciplines. Across the current 2026 abstracts, recurring themes include translation of mechanistic discovery toward patient care, diagnostic vigilance in uncommon presentations, attention to social and global determinants of health, learner-driven educational innovation, and quality improvement designed to make care more reliable.

## TRANSLATIONAL AND BASIC SCIENCE INNOVATION

The translational science contributions move from developmental and regenerative biology to cancer biology, molecular trafficking, imaging, and neurocellular mechanisms. Studies using zebrafish, human organoid and assembloid systems, tumor models, extracellular vesicles, and advanced imaging show how mechanistic experimentation can be linked to clinically meaningful questions. Rather than separating basic science from patient care, these abstracts position target discovery, model refinement, and measurement technologies as foundations for future therapeutic and diagnostic advances.^1–9^

Representative contributions include O’Hern et al on “Human Heart-Macrophage Assembloids Mimic Immune-Cardiac Interactions and Enable Arrhythmia Modeling”; Tsurho et al on “Sensitizing Temozolomide-resistant Glioblastoma to Ferroptosis with High-dose L-ascorbic acid”; Mannering et al on “Human Heart–Macrophage Assembloids Capture Tissue-Resident Immune Integration During Cardiac Development”; Bonacci et al on “RSAD2-CMPK2 Signaling Mediates PVT1 Exon 9 Action in an AR-Independent Manner in PVT1 Exon 9 Overexpressing Neuroendocrine Prostate Cancers”.

## CARDIOVASCULAR, VASCULAR, PULMONARY, AND CRITICAL CARE

Cardiovascular and acute-care investigations form a prominent thread, spanning pulmonary arterial hypertension, atrial fibrillation, coronary syndromes, mechanical circulatory support, thrombosis, pulmonary embolism, resuscitation, and vascular catastrophe. Collectively, they emphasize vigilance across the continuum of care: established therapies and devices can improve outcomes, yet uncommon complications and atypical presentations often require rapid integration of imaging, physiology, bedside examination, and multidisciplinary judgment.^1,10–30^

Representative contributions include Xu et al. on “Cardiomyocyte Proliferation in Embryonic Zebrafish Hearts Induced by Novel Small-Molecule Compounds”; Fatima et al. on “Efficacy and Safety of Sotatercept in Pulmonary Artery Hypertension: A Meta-analysis of Randomized Control Trials”; Chopra et al. on “Severity Matters: Inpatient Outcomes of Atrial Fibrillation Across The Spectrum of Gastroesophageal Reflux Disease”; Goldberg et al. on “Resuscitation Systems Analysis in Botswana Using the Global Frame of Survival: A Narrative Review”.

## NEUROLOGY, OPHTHALMOLOGY, AND REHABILITATION

Neurologic, neuro-ophthalmic, ophthalmologic, and rehabilitation reports repeatedly demonstrate the value of serial examination when imaging or initial diagnostic labels are incomplete. Work on concussion, intracranial pressure, retinal disease, encephalopathy, stroke and stroke mimics, spinal and demyelinating disorders, and medication-associated vestibular dysfunction reinforces a practical principle: diagnostic certainty often emerges longitudinally through repeated bedside assessment and reconciliation of clinical findings with imaging and laboratory data.^31–59^

Representative contributions include Liebenthal et al. on “Longitudinal Changes in Sports-Related Concussions Across the COVID-19 Pandemic: An Interrupted Time Series Analysis”; Kumar et al. on “Discrepancies Between Automated and Manually Corrected RNFL Measurements in Papilledema Associated with Idiopathic Intracranial Hypertension”; Bischel et al. on “Clonal Hematopoiesis as a Predictor of Immune Toxicity and Efficacy After Chimeric Antigen Receptor T-cell Therapy: a Systematic Review and Meta-Analysis”; Nesheiwat et al. on “Validation of Brain Injury Guidelines in the Elderly Trauma Patient Presenting at a Level Two Trauma Center”.

## INFECTIOUS DISEASE, IMMUNOLOGY, AND GLOBAL HEALTH

The global health and infectious disease portfolio is notably broad. Population-based studies from the Democratic Republic of Congo, Peru, the Dominican Republic, Guatemala, Botswana, and Michigan examine nutrition, mental health, sexual health, chronic disease, vaccination, and health-system access, while case reports address tuberculosis, leishmaniasis, invasive bacterial disease, nontuberculous mycobacteria, Lyme disease, and fungal infection. Together, they demonstrate that geography, social determinants, exposure history, and access to care can be as diagnostically consequential as laboratory testing.^20,55,60–79^

Representative contributions include Koduri et al. on “Household Food Insecurity and Parenting Confidence and Stress among Women in the Democratic Republic of Congo”; Lossada-Soto et al. on “Age at Sexual Debut and High-Risk Sexual Behavior in Association to HPV Risk in Two Regions of Peru”; Kankanalapalli et al. on “Mental Health and Behavioral Symptoms Among Children Living in Democratic Republic of Congo, by Sex and Age”; Dunmore et al. on “Quality of Life Assessment of Post-Cholecystectomy Patients in Iquitos, Perú”.

## MEDICAL EDUCATION, QUALITY IMPROVEMENT, AND HEALTH SYSTEMS

Education and systems-improvement studies demonstrate scholarship directed at the learning environment and the reliability of care. Topics include electronic health record templates, learner anxiety and preparation, board-examination resources, adolescent health education, pipeline programs, musculoskeletal competency, medication-administration training, tele-supervision, and graduate medical education. These projects reinforce the role of residents and students not only as learners but also as investigators who can redesign workflows and measure educational and clinical outcomes.^80–93^

Representative contributions include Lotfi et al. on “Perceptions Regarding Proposed Changes to ACGME Program Requirements for Emergency Medicine”; Kanning on “Sustained Effects of Multi-Modal Educational Interventions on Pediatric Emergency Department Utilization Patterns”; Vora et al. on “Impact of a Resident-Built, ADA-Aligned Epic Template on Guideline-Based Diabetes Care Processes and HbA1c in a Teaching Clinic: A Quality Improvement Initiative”; Saleh et al. on “Frailty Risk Model for Readmission after Transoral Robotic Surgery for Oropharyngeal Squamous Cell Carcinoma”.

## MUSCULOSKELETAL MEDICINE, SURGERY, PAIN, AND OSTEOPATHIC CARE

Musculoskeletal, surgical, pain, and osteopathic contributions range from pelvic fixation and casting safety to chronic wound analgesia, peri-procedural pain management, scar treatment, osteopathic manipulative approaches, and unusual postoperative or musculoskeletal complications. Several reports connect structural assessment with function and recovery, reflecting the osteopathic emphasis on anatomy, mobility, and whole-person care while subjecting interventions to measurable outcomes.^94–105^

Representative contributions include Juma et al. on “Development of the Pelvic Reduction Grading System (PRGS): A Radiographic Tool for Assessing Alignment After Pelvic Ring Fixation”; Kepes et al. on “Treatment of C-Section Scar with Light Touch as a Comparator to Myofascial Release in Patients with Chronic Low Back Pain Case Report”; Heliin et al. on “Physical Medicine & Rehabilitation Physicians’ Confidence Identifying Opioid Misuse Following CDC 2016 Prescribing Guidelines”; Myers et al. on “Topical Opioids for Pain in Malignant and Non-Malignant Chronic Ulcers: A Systematic Review”.

## ONCOLOGY, DERMATOLOGY, ENDOCRINE, RENAL, AND MULTISYSTEM MEDICINE

A diverse group of oncology, dermatology, endocrine, renal, and multisystem reports addresses biomarker-guided care, malignancy, medication toxicity, metabolic disease, autoimmune-appearing presentations, and rare pathologic findings. These contributions underscore phenotypic overlap: benign and malignant processes may mimic one another, adverse drug effects may resemble primary disease, and serologic or radiographic findings may point in competing directions. Pathology, medication history, and longitudinal follow-up therefore remain essential anchors of diagnostic reasoning.^106–119^

Representative contributions include Mantravadi et al. on “Benign Metastasizing Leiomyomatosis: The Paradox of a Benign Tumor That Spreads”; Darboe et al. on “CD44 as a Predictive Biomarker in Non-Small Cell Lung Cancer Patients Treated with Radiotherapy”; Gopannagari et al. on “Differential Survival Gains Across Histologic Subtypes of Metastatic Renal Cell Carcinoma Across Therapeutic Eras: A SEER 2006–2022 Analysis”; Baj-Osiewicz et al. on “Bullous Pemphigoid Development in a Patient Treated with Dulaglutide: A Case Report”.

## DIAGNOSTIC DILEMMAS, ADVERSE EFFECTS, AND UNCOMMON CLINICAL PRESENTATIONS

The remaining case-based scholarship is unified less by organ system than by diagnostic uncertainty. These reports describe uncommon adverse effects, delayed diagnoses, misleading initial tests, unusual anatomic findings, and rare presentations of familiar diseases. Their educational value lies in disciplined reconsideration: when a patient fails to follow the expected clinical course, the differential diagnosis must reopen and apparently reassuring tests must be interpreted within the full clinical context.^66,110–130^

Representative contributions include Whitman et al. on “Sharing the View: Obtaining and Sharing Slit-Lamp Microscopy High Quality Images.”; Kam et al. on “Directed or Random? Student Reasoning About Diffusion Across Physiological Contexts”; Cross et al. on “Extended Focused Assessment with Sonography Accuracy at a Community Level III Trauma Center”; Abbas et al. on “Pancreatic Pseudocysts with Progressive Multiorgan Dysfunction”.

## CROSS-CUTTING THEMES: DIAGNOSTIC HUMILITY, TEAM SCIENCE, AND LEARNER SCHOLARSHIP

Across the 2026 contributions, three features stand out. First is diagnostic humility: many reports begin with a plausible initial diagnosis that becomes insufficient when symptoms persist, new data emerge, or the patient fails to respond as expected. Second is team science. Author groups repeatedly bring together students, residents, fellows, faculty, and investigators from different disciplines and institutions. Third is the visibility of trainees as first authors and project leaders. In a journal closely connected to graduate medical education, scholarly participation is not merely an academic requirement; it is a mechanism for teaching critical appraisal, scientific communication, systems thinking, and reflective clinical practice.

## THE VALUE OF ABSTRACT SCHOLARSHIP

Abstract collections occupy a distinct place in the scientific record. They capture work at an earlier stage than many full-length articles, preserving the immediacy of new observations, pilot data, quality-improvement cycles, and educational innovations. Their limitations are equally important: abbreviated methods and results constrain appraisal, many findings require replication or fuller reporting, and case reports primarily generate clinical awareness rather than causal inference. Read together, however, these abstracts provide a valuable map of emerging questions and of the clinical problems trainees and faculty encounter in practice.

For SMRJ, the special issue therefore serves two complementary purposes. It disseminates new findings while documenting a culture of inquiry across affiliated institutions. The range of methods—from molecular models and meta-analysis to retrospective database studies, surveys, quality-improvement interventions, point-of-care imaging, and detailed case observation—demonstrates that meaningful scholarship can arise from many settings when the research question is clear and interpretation remains proportionate to the evidence.

## CONCLUSION

Taken together, the 2026 abstracts portray a learning health community in which discovery, clinical observation, education, and systems improvement are mutually reinforcing. The strongest contributions do more than report an unusual finding or a statistically significant association: they clarify a clinical problem, expose an uncertainty, test a practical intervention, or identify a question that deserves deeper investigation. By bringing these diverse forms of scholarship into a single issue, SMRJ provides a forum in which early scientific discovery, bedside reasoning, educational innovation, and population health can inform one another.

### Corresponding Author

Francis O. Akenami, BMLS, PhD, FIMLS

Managing Editor - Spartan Medical Research Journal

Research, Innovation, and Scholarly Engagement (RISE)

Michigan State University, College of Osteopathic Medicine

Email: akenamif@msu.edu
